# On Dentine

**Published:** 1877-04

**Authors:** Chas. Sibler


### ARTICLE VIII.

### *On Dentine.*

BY CHS. SIBLER, M. D.

I. Proposition. Dentine as it exists in the living and healthy tooth consists of three kinds of substances:

First. A homogeneous, glue like substance in which the lime salts are deposited. On this substance, together with the lime-salts, depends the hardness of the tooth.

Second. A material of a similar character as that of which the yellow elastic fibre (ligamentum nuchae periosteum) is made. This exists in the form of tubes forming the lining of the dentinal canals with their side branches, and gradually merging into the odontoblast. On this material, I think, the elasticity of the tooth depends.

Third. The germinal matter which fills out the tubules and makes up the main portion of the odontoblasts.

By the term germinal matter is meant the living or forming substance which has the power to take up nourishment and transform it into tissue substances.

II. Proofs. First. Take a longitudinal section through the root of a tooth and examine it dry under the microscope. You will see black lines running from the pulp cavity out wards. What do these black lines signify?

Second. Add some turpentine to the section. You can see it enter the places or spaces that appeared as dark lines, and as the air is expelled the section takes on a homogeneous appearance, The turpentine then has proved that there are canals (not tubes) in dentine.

Third. Decalcify a thin section of dentine which presents the dentinal canals longitudinally, with dilute muriatic acid, and then treat it with strong muriatic acid and watch it under the microscope. The material between the canals will swell up at first and then grow soft, finally disappear and there will remain, resisting the action of the strong acid for some time white glistening fibers, apparently, corresponding to the canals of the dentine. Are these fibers or tubes?

Fourth. Make such a section of dentine as will cut the canals transversely, or a longitudinal section which will give you a view of the pulp cavity and the openings of the dentinal canals. Now you will be able to see that these resisting elements are tubular.

Fifth. Take an incisor tooth of a calf, and split off a piece of the root, being careful to disturb as little as possible the connection of the pulp and the dentine in the remaining portion of the dentine, and then stain the tooth by leaving it in Beale's carmine for fourteen days and longer. You will then frequently see the contents of the tubes and the odontoblasts stained, proving that there is germinal matter contained in the tubes.

Sixth. After making sections through the dentine and pulp attached, and then adding a little acetic acid to such a section and pressing it with the covering glass, it will happen that fibers *appear* stretched from the pulp which has been pushed off a distance from the dentine, across the interval and entering the dentine, and it can be seen, further that these elements grow thinner the more they are stretched and the wider the interval between the pulp and dentine.

Seventh. Again, it will happen when the pulp is separated from the dentine, that numerous short processes stand out from the pulp corresponding in thickness to the dentinal canals and having a granular appearance.

Eighth. When a recently pulled tooth with the pulp in it is decalcified, and sections, say through the root, are made parallel to the tubules and the pulp attached, it will be seen, when such a section is treated with strong muriatic acid, that the remaining tubules do not end at the dentine, but are in connection with the pulp.

Ninth. When these sections are made with a hard knife showing the dentinal canals with odontoblasts in front of and connection with them, treatment with muriatic acid as above will show that the odontoblasts and the dentinal tubules are in continuity.

Eight and nine show that the tubules with their contents and the odontoblasts are in continuity and in a certain sense might be considered a unit.

Six and seven show that the fibers are not a soft fibril or germinal matter alone, for how could this semi-fluid material stand so much stretching. And that there is something of a tubular nature present is shown by the circumstance that the more these fibers are stretched the smaller their caliber.

Taking together the granular appearance, and the circumstance that the fibres can be thus stretched, and that the odontoblasts, which by staining have been proven to consist of germinal matter, are in direct continuity with the tubules and the contents of these, which also by staining have been proven to contain germinal matter, and that muriatic acid makes such a marked distinction between the tubular and the intertubular substance, the proposition will I think, have support from facts.

The view here presented, on the structure of dentine, will agree with that of Beale, if Beale admits that his formed matter consists of two chemically distinct substances, just as distinct as the walls of the fat vesicle and the fat contained in it, and if Beale admits that this acid resisting material is produced *pari passu* with the homogeneous interbular material.

This view will agree with that of Tomes, if Tomes admits that his soft fibril is not so very soft after all, and that the dentinal tube and the outer portion of his soft fibril are one and the same thing; then the semi-fluid which Tomes has observed protruding from torn fibrils will agree with our germinal matter within the tubes.

This view will agree with that of Koelliker, Waldeyer and others, if they admit that, "their" dentinal fibrils and dentinal (Neumann's) sheaths are one and the same thing.

This view will agree with that of Salter, if Salter will admit, that his thick fluid in the tubes, is a substance which deserves a better name, and if Salter will admit as not proven that the dentinal tubules are calcified.—*Dental Register*.

